# A Systematic Review of Urban Navigation Systems for Visually Impaired People

**DOI:** 10.3390/s21093103

**Published:** 2021-04-29

**Authors:** Fatma El-zahraa El-taher, Ayman Taha, Jane Courtney, Susan Mckeever

**Affiliations:** 1 School of Computer Science, Technological University Dublin, D07EWV4 Dublin, Ireland; fatma.e.eltaher@mytudublin.ie (F.E.-z.E.-t.); ayman.farahat@tudublin.ie (A.T.); jane.courtney@tudublin.ie (J.C.); 2 Faculty of Computers and Artificial Intelligence, Cairo University, Cairo 12613, Egypt

**Keywords:** assistive systems, navigation systems, visually impaired people, smart cities, planning journeys, independent children navigation, obstacle avoidance, autonomous driving, robot navigation

## Abstract

Blind and Visually impaired people (BVIP) face a range of practical difficulties when undertaking outdoor journeys as pedestrians. Over the past decade, a variety of assistive devices have been researched and developed to help BVIP navigate more safely and independently. In addition, research in overlapping domains are addressing the problem of automatic environment interpretation using computer vision and machine learning, particularly deep learning, approaches. Our aim in this article is to present a comprehensive review of research directly in, or relevant to, assistive outdoor navigation for BVIP. We breakdown the navigation area into a series of navigation phases and tasks. We then use this structure for our systematic review of research, analysing articles, methods, datasets and current limitations by task. We also provide an overview of commercial and non-commercial navigation applications targeted at BVIP. Our review contributes to the body of knowledge by providing a comprehensive, structured analysis of work in the domain, including the state of the art, and guidance on future directions. It will support both researchers and other stakeholders in the domain to establish an informed view of research progress.

## 1. Introduction

According to the World Health Organization (WHO), at least 1 billion people are visually impaired in 2020 [1]. There are various causes of vision impairment and blindness, including uncorrected refractive errors, neurological defects from birth, and age-related cataracts [1]. For those who suffer from vision impairment, both independence and confidence in undertaking daily activities of living are impacted. Assistive systems exist to help BVIP in various activities of daily living, such as recognizing people [2], distinguishing banknotes [3,4], choosing clothes [5], and navigation support, both indoors and outdoors [6].

BVIP face particularly serious problems when navigating public outdoor areas on foot, where simple tasks such as crossing a road, obstacle avoidance, and using public transportation present major hazards and difficulties [7]. These problems threaten the confidence, safety and independence of BVIP, limiting their ability to engage in society. In recent years, technological solutions to support BVIP in outdoor pedestrian navigation has been an active research area (see Table 1). In addition, we find that overlapping areas of research, whilst not tagged as assistive navigation systems research, are addressing challenges that can contribute to its progress, such as smart cities, robot navigation and automated journey planning. The combined substantial body of work needs further examination and analysis in order to understand the progress, gaps and direction for future research towards full support of BVIP in outdoor navigation. Our review provides a comprehensive resource for other researchers, commercial and not for profit technology companies, and indeed to any stakeholders in the BVIP sector.

The contributions of this survey are summarised as follows:

1. A hierarchical taxonomy of the phases and associated task breakdown of pedestrian urban navigation associated with safe navigation for BVIP, is presented.
2. For each task, we provide a detailed review of research work and developments, limitations of approaches taken, and potential future directions.
3. The research area of navigation systems for BVIP overlaps with other research fields including smart cities, automated journey planning, autonomous vehicles, and robot navigation. We highlight these overlaps throughout to provide a useful and far-reaching review of this domain and its context to other areas.
4. We highlight and clarify the range of used terminologies in the domain.
5. We review the range of available applications and purpose-built/modified devices to support BVIP.

In this survey, we mainly included papers that discussed the area of outdoor navigation systems for BVIP from 2015 until 2020. The paper comprises recent scientific works to reveal the current gaps and future trends of the area. However, sometimes we encompass papers from earlier years if it has significant information. We used Google Scholar as a source of papers. Firstly, we searched for assistive and aid navigation systems for VI. Secondly, for each task, we used different keywords to look for the scientific works which related to the area of interest that we are concerned with. In addition, we checked work that was done within our domains. Finally, we excluded papers under two criteria (1) if a paper is irrelevant after reading the abstract, or (2) if a paper is published in journals and conferences with an impact factor of less than one.

The structure of our review is as the following. Section 2 discusses previous surveys in the area of outdoor navigation for BVIP, and explored different terminologies in the area. The taxonomy of phases and tasks of assistive navigation systems is presented in Section 3. In Section 4, the analysis of previous research works in assistive outdoor navigation systems for BVIP is explored. Section 5, Section 6 and Section 7 explore each phase and its tasks in detail, including both BVIP research and overlapping domain research for each task. We explore other aspects of designing navigation systems such as feedback and wearability in Section 8. In Section 9, applications and devices are compared. Section 10 summarizes the main findings of our review and discusses the main challenges in the area. Finally, a conclusion and future work are highlighted in Section 11.

## 2. Related Work

Our focus in this section is to examine the range and scope of previous reviews in the domain of navigation system for the BVIP domain. Islam et al. [8] focussed specifically on walking systems. They compared indoor and outdoor walking systems that support BVIP during navigation. To conduct this comparison, they used the following features: capturing devices, feedback devices/types, hardware components, coverage area, detection range, weight, and cost-effectiveness. Real and Araujo [9] presented a historical development of indoor and outdoor navigation systems between 1960 and 2019. However, they did not discuss the underlying algorithms used.

Fernandes et al. [10] defined the main components in navigation systems—namely interface, location, orientation, and navigation. They also presented a review of technologies that were used for each component. They emphasized the need to combine various technologies together to build a comprehensive system. Their review, however, did not study in detail the algorithms and datasets and did not attempt to present a comparison between systems. Paiva and Gupta [11] explored indoor and outdoor navigation systems and obstacle detection systems. They identified approaches and equipment used in each one. However, they excluded a comparison between approaches and a discussion about the algorithms used.

A number of reviews presented small-scale surveys of a small number of indoor and outdoor navigation systems [12,13,14]. While they provided information about technologies and limitations, they did not mention or explore the applied algorithms. Manjari et al. [15] explored previous navigation systems in the domain and defined features of each one. They provided a brief and general summary of utilized algorithms and techniques but did not provide detailed analysis of data, techniques, methods or gaps.

Tapu et al. [16] assessed features of outdoor navigation systems such as wearability, portability, reliability, low cost, real-time, user-friend, robustness, and wireless/no connection. Although they presented a new direction of evaluation Electronic Travel Aids (ETAs), they covered only 12 articles.

### 2.1. Specific Sub-Domain Surveys

Survey publications in this category have explored navigation systems for a specific sub-domain—where they have discussed the previous work from one perspective, such as computer vision.

Fei et al. [17] focused on indoor and outdoor ETAs based on computer vision. They classified ETAs according to the provided information to the user during the journey, classifying by road situations and obstacles, reading signs and tags, object recognition, and text extraction. The features and limitations of each system were explained. However, they did not discuss the future work of ETAs or compare between available systems. Budrionis et al. [18] compared 15 mobile navigation applications that use computer vision. A comparison was done from distinct perspectives (objectives/functions, input/output, data processing, algorithms, and evaluation of the solution). The capabilities of a smartphone to help BVIP in their navigation are discussed by Kuriakose et al. [19]. They identified the advantages and limitations of six smartphone applications [19]. Budrionis et al. [18] and Kuriakose et al. [19] included a limited number of navigation systems. This lack of included articles eliminates use of these surveys as a complete overview of the area.

To recap, no single review provides a complete and detailed coverage of research into navigation supports for the BVIP sector. The majority of previous surveys reviewed a limited number of published works, resulting in either a narrow or a more cursory presentation of previous work. Likewise, previous reviews discussed navigation systems at a high level, without including details about how the individual aspects or tasks of navigation were addressed. In addition, the algorithms and associated research datasets were not discussed, so state of the art approaches and the existence of benchmarks datasets are not identifiable. As a result, the previous review articles present a cursory overview of an area of interest. This lack of a comprehensive in-depth review of this domain motivated us to investigate this area and present our survey.

### 2.2. Terminology

This subsection will present the different terminologies used in a navigation systems for the BVIP community. In addition, it emphasizes that there is no agreed terminology. There are five phrases used to express all activities related to navigation of BVIP, namely walking assistants for BVIP [8], traveling aid systems for BVIP [20], visual substitution navigation systems for BVIP [21], navigation systems for BVIP [9], and assistive navigation systems for BVIP [10]. In addition to these different terms, navigation activities are classified in different ways and have various meanings. Traveling aid system tasks were divided into micro-navigation tasks (define obstacles and the environment around the user) and macro-navigation tasks (related to defining a path to a destination and information needs like the existence of intersections, road signs, and so on) [20].

Fernandes et al. [10] defined the required tasks for assisting people in navigation. These tasks are (1) an interface (to convey useful information to a user) (2) localization (to define the location of the user) (3) orientation (to define the environment around the user) and (4) navigation (to define the route for the destination). Dakopoulos and Bourbakis [21] divided the visual substitution systems for navigation to (1) ETAs: to receive data about surroundings, such as obstacles, (2) Electronic Orientation Aids (EOAs) which help the user to reach a destination by selecting the route, and (3) Position Locator Devices (PLDs) which defines the user’s location.

The definition of travel aids differs somewhat across the research. For example, Petrie et al. [20] considered a travel aid to be a system that involves all tasks related to navigation activities. On the other hand, Manjari et al. [15] define travel aids as responsible only for understanding the environment. The term “orientation” is used with two different definitions. The absence of agreed terminology can lead difficulties in understanding literature, especially for new readers in the area. In addition, it may lead to the investigator accidentally excluding research works using these different terms during searching.

## 3. A Taxonomy of Outdoor Navigation Systems for BVIP

**Figure 1:**
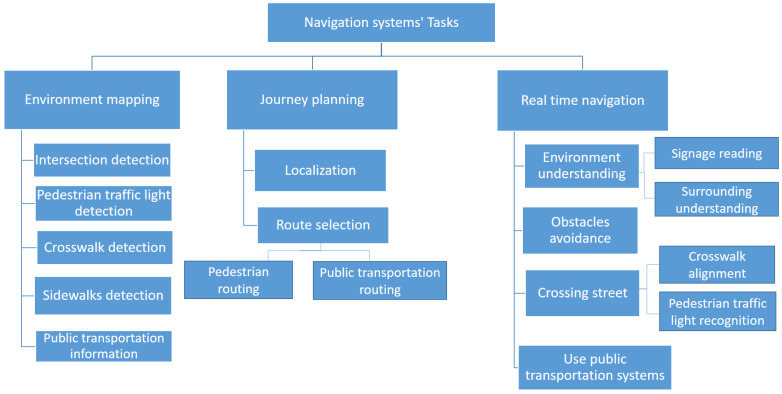
A taxonomy of navigation support tasks for BVIP pedestrians.

Assistive navigation systems in an urban environment focus on any aspect of supporting pedestrian BVIP in moving in a controlled and safe way for a particular route. The first step in analysing this domain is to develop and apply a clear view on both the scope and terminology involved in outdoor pedestrian navigation systems. We present a taxonomy of outdoor navigation in Figure 1. At the top level, we identify the three main sequenced phases which encompass the area of outdoor navigation systems, from environment mapping, through journal planning to navigating the journey in real time. Each of these phases consists of a task breakdown structure. The tasks comprise the range of actions and challenges that a visually impaired person need to succeed at in order to move successfully from an initial point to a selected destination safely and efficiently. In effect, the phases represent higher-level research areas, while the task breakdown structure for each phase shows the research sub-domains.

Looking at each phase in Figure 1, the *environment mapping* phase provides appropriate and relevant location-specific information to support BVIP pedestrians in journey planning and real-time journey support. It defines the locations and information of static street elements such as intersections, public transportation stations, and traffic lights. The environment mapping phase is an off-line up-front data gathering and processing phase that underpins the remaining navigation phases. The second phase *Journey planning* begins by determining the start location. It then selects the optimal route to the user’s destination, allowing for safety and routing, using the information from the environment mapping phase. Finally, BVIP need support for challenges in *real-time navigation* including real-time environment understanding, crossing a street, obstacle avoidance, and using public transportation. We explain each of the taxonomy entries in more detail:

**Environment mapping phase:**

Existing map applications do not provide the level of information needed to support the BVIP community when planning and undertaking pedestrian journeys. This phase addresses the tasks associated with enriching available maps with useful information for such journeys. Pre-determined location and information about sidewalks, public transportation, road intersections, appropriate crossing points (crosswalks), and availability of traffic lights are all essential points of information for this user group. We identify five tasks or sub-domains within the environment mapping phase.

- *Intersection detection:* detects the location of road intersections. An intersection is defined as a point where two or more roads meet, and represents a critical safety point of interest to BVIP.
- *Pedestrian traffic light detection:* detects the location and orientation of pedestrian traffic lights. These are traffic lights that have stop/go signals designed for pedestrians, as opposed to solely vehicle drivers.
- *Crosswalk detection:* detects an optimal marked location where visually impaired users can cross a road, such as a zebra crosswalk.
- *Sidewalk detection:* detects the existence and location of the pedestrian sidewalk (pavement) where BVIP can walk safely.
- *Public transportation information:* defines the locations of public transportation stops and stations, and information about the degree of accessibility of each one.

**Journey planning phase:**

For the BVIP community, journey planning is a critical part of building the confidence and knowledge to undertaking a pedestrian journey to a new destination. This phase supports the planning of journeys, so as to select the safest and most efficient route from a BVIP’s location to their destination. It builds upon the enriched mapping information from the environmental mapping phase, and consists of the following two tasks:

- *Localization:* defines the initial start point of the journey, where users start their journey from.
- *Route selection:* finds the best route to reach a specified destination.

**Real-time navigation phase:**

The final phase is about supporting the BVIP while undertaking their journey. Real-time navigation support recognises the dynamic factors during the journey. We identify the following four tasks or sub-domains:

- *Environment understanding:* helps BVIP to understand their surroundings, including reading signage and physical surrounding understanding.
- *Avoiding obstacles:* detects the obstacles on a road and helps BVIP to avoid them.
- *Crossing street:* helps BVIP in crossing a road when at a junction. This task helps the individual to align with the location of a crosswalk. Furthermore, it recognizes the status of a pedestrian traffic light to determine the appropriate time to cross, so they can cross safely.
- *Using public transportation systems:* This task assists BVIP in using public transportation systems such as a bus or train.

In the next section, we provide a snapshot of the navigation systems research published, mapped against the tasks in our taxonomy. This will establish the extent of research in the BVIP navigation system domain, and the focus of this research in relation to the tasks presented in our taxonomy. We noted earlier that many tasks represent a sub-domain of research in themselves, and are addressed by research works from a variety of application domains. We provide a detailed analysis of the research against each phase/task in Section 5, Section 6 and Section 7 so as to capture both BVIP and relevant non BVIP work. For each task, we present the state-of-the-art, overlaps with other areas, gaps in the research approaches taken to date, and directions for future work.

## 4. Overview of Navigation Systems by Device

Navigation systems research literature differs substantially along two particular lines (1) the scope and depth of the functionality (akin to tasks) offered across these systems and (2) the nature of the hardware/device provided to the user, which gathers (perceives) data about the environment. This data may be a captured image or other sensor feedback. Navigation assistive systems extract useful information from this data to help the BVIP during their navigation—such as the type and location of obstacles. We divide assistive systems for BVIP into four categories, based on the used device for data gathering:

- Sensors-based: this category collects data through various sensors such as ultrasonic sensors, liquid sensors, and infrared (IR) sensors.
- Electromagnetic/radar-based: radar is used to receive information about the environment, particularly objects in the environment.
- Camera-based: cameras capture a scene to produce more detailed information about the environment, such as an object’s colour and shape.
- Smartphone-based: in this case, the BVIP has their own device with a downloaded application. Some applications utilise just the phone camera, with others using the phone camera and other phone sensors such as GPS, compass, etc.
- Combination: in these categories, two types of data gathering methods are used to combine the benefits of both of them such as sensor and smartphone, sensor and camera, and camera and smartphone.

To establish a broad-brush view of the BVIP specific literature in BVIP systems, we present Table 1.

Research works are classified across the phases/tasks of navigation systems and the type of device/hardware system, as shown in Table 1. From this table, we note that the tasks that have received the most attention from the research community are the tasks of obstacle avoidance and localization. Secondly, while the environment mapping phase is a critical part of BVIP navigation systems, it is has not been addressed in the navigation systems for BVIP research base so is not included here. Thirdly, we note that previous navigation systems work has not included signage reading as a focus area, with just two published work. Although using public transportation systems has a significant effect on the mobility and employment of BVIP, it is not included in the majority of navigation systems. None of the previous articles address all tasks for real-time navigation, so no single system presents a complete navigation solution to the BVIP community. We note that while most hardware/device systems aim to address aspects of both journey planning and real-time navigation, sensor and camera based systems focus solely on the tasks of obstacle avoidance. In addition, there is only one smartphone based system that uses a separate camera in the literature suggesting that smartphone solutions rely on the in-built camera.

Having examined the distribution of BVIP navigation systems research effort across navigation functions, we now analyse the research base at a more detailed level using our phase and task taxonomy. As our focus is by task, we include both BVIP and non BVIP literature.

**Table 1:** Tasks coverage of published navigation systems, by data collection device.

Devices	Journey Planning		Real-Time navigation					
	Localization	Route Selection	Environment Understanding		Obstacle Avoidance	Crossing Street		Using Public Transportation
			Signage Reading	Surrounding Understanding		Pedestrian traffic Lights Recognition	Crosswalk Alignment	
Sensors-based	[22,23,24]				[22,23,25,26,27,28,29,30,31]			[32]
Electromagnetic/radar-based					[33,34,35]			
Camera-based	[36,37,38,39]		[40,41]	[42,43]	[39,44,45,46,47,48,49,50]	[51,52,53]	[51]	
Smartphone-based	[54,55,56,57,58,59]	[54,55,57]		[57,58]	[57,60,61]	[57,62,63,64]	[62,65]	[66]
Sensor and camera based					[67,68,69,70,71]			
Electromagnetic/radar-based and camera based					[72]			
Sensor and smartphone based	[73,74,75]	[74]			[73,75,76]	[77]	[77]	[78,79]
Camera and smartphone based	[80]	[80]			[80]			

## 5. Environment Mapping

The first phase of navigation systems is an environment mapping phase. This phase is about converting street elements to practical information on maps. There are a large variety of permanent and semi-permanent street components that are relevant to BVIP, including intersections, traffic lights, crosswalks, transportation stations/stops and sidewalks. Whilst these safety-critical components are easy to detect by sighted people, they present a huge challenge for BVIP—with environment mapping representing a fundamental phase in navigation systems that has limited attention thus far in the research domain. This encourages us to study work done on other domains to determine the research challenges and gaps as well as introduce prospective future directions on the environment mapping phase detailed by task. As a result, this emphasizes the need to transfer knowledge between other domains and the area of navigation systems for BVIP.

### 5.1. Intersection Detection

The intersection detection task is an important component of an environment mapping stage as it helps BVIP to avoid uncontrolled intersections on their journey (i.e., those that do not have traffic lights). Previous research works used different ways to recognize junctions, such as the existence of traffic lights [53,62], audible units [77], or ramps [81].

**Figure 2:**
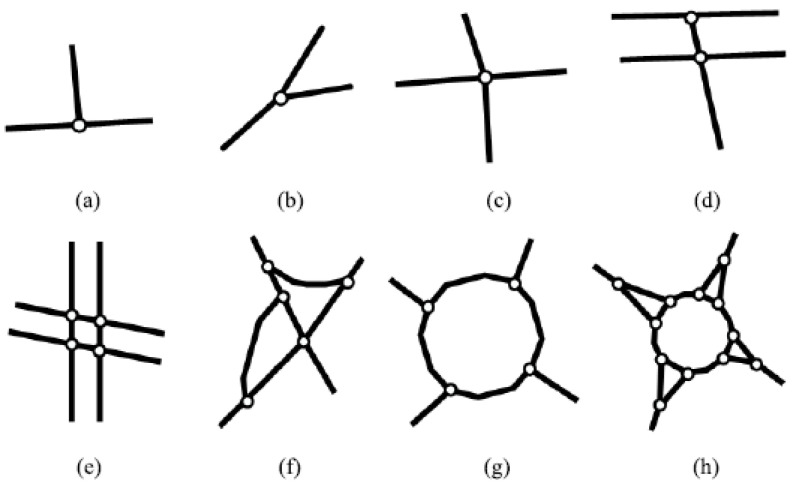
Types of intersection from Dai et al. [83]: (**a**–**c**) typical road intersections; (**d**–**f**) complex intersections; (**g**,**h**) round-about intersection

Both the existence and type of intersection are important to the BVIP, as the type will determine how the road should be navigated. Intersection types vary across the literature. Zhou and Li [82] identified nine types of intersections. Dai et al. [83] classified junctions into three main classes: the typical road intersection structure (Figure 2a–c), the complex typical intersection structure (Figure 2d–f), and the round-about road intersection structure (Figure 2g,h), as shown in Figure 2.

By analysing the various types of intersections, we found that there are 14 unique types of junctions. We also note that intersection detection task is discussed in several domains such as autonomous vehicles [84], driver assistance systems [85], and transformation of maps to digital datasets [86]. Although it is significant for navigation systems [7], it is not addressed in any of them.

A variety of data sources are used in the detection of intersections: images [87], map tiles [86], videos [88], LiDAR sensors [85], and vehicle trajectories [89,90]. Here, computer vision approaches will be discussed as images and videos are considered a rich source of information, providing detailed junction information, such as the number of lanes. The problem of intersection detection has been addressed to date via two computer vision approaches:

*An image classification problem:* researchers have treated the problem as three levels of classification: a binary problem of existence of an interface, a multi-class intersection type problem, and a road detection problem. This latter approach is about detecting a road in an image, and then determining intersections as part of road detection [87,91]. Looking at each in turn, for *binary classification:* Kumar et al. [88] determined the existence of an intersection in a video or not—the network consists of Convolutional Neural Network (CNN), bi-Long short-term memory (LSTM), and Siamese-CNN. For BVIP, however, the type of intersection is also important, so this approach has limited use. Looking at the problem as *a multi-classification* intersection type problem, Bhatt et al. [84] used CNN and LSTM networks to classify sequences of frames (video) into three classes non-intersection, a T-junction, a cross junction. Oeljeklaus et al. [92] utilized a common encoder for semantic segmentation and recognition of road topology tasks. They were able to recognize six types of intersections. Koji and Kanji [93] used two types of input. First, they used images before an intersection of Third-Person Vision (TPV) and sequences of images while an intersection is passed First-person vision (FPV). For TPV, they used deep Convolutions Neural Networks (DCN) and applied LSTM for FPV. Finally, they integrated the two outputs to define seven classes of junctions. The third approach, *identify road before classification*, both Rebai et al. [91] and Tümen and Ergen [87] depend on different edge-based approaches to detect the road prior to the classification step. For a classification step, Rebai et al. [91] used a hierarchical support vector machine (SVM), while Tümen and Ergen [87] applied a CNN network.

*An object detection problem:* Saeedimoghaddam and Stepinski [86] dealt with an intersection detection task as an object detection problem, detecting both the existence and placement of the intersection within the scene (image). They used Faster RCNN to define all intersections on map tiles, achieving an 0.86 F1-score for the identification of road intersections.

*Datasets in intersection detection research:* Researchers may wish to use existing datasets for comparative evaluations or to support model developments. The datasets used in intersection detection model training and testing are listed in Table 2.

### 5.2. Pedestrian Traffic Lights Detection

Pedestrian Traffic Lights (PTLs) are an essential component of an urban environment. Thus, defining the location of PTLs is an important part of the environment mapping phase. The existence of PTLs is mandatory for crossing roads, but is particularly critical for the BVIP community [62]. Selection of the safest route should exclude all uncontrolled intersections. Recently, the detection and geolocation of different street objects from street images, such as traffic lights, were discussed [100,101]. This line of research which enables automatic mapping of complex street scenes with multiple objects of interest is in the general domain of street object identification will be of interest to the BVIP research community as the importance of environment mapping becomes apparent. However, location needs to be captured for environment mapping in order to provide rich mapping information.

### 5.3. Crosswalk Detection

Highlighting designated crosswalk locations is an important task in an environment mapping phase. Adding this type of information will support better route selection to include designated crosswalks where people can cross safely [51]. While this is considered a simple task for sighted people, it is a challenging one for BVIP, whereby they must understand where the crosswalk is, and also the placement of the crosswalk on the street, so that the BVIP crosses within the boundaries of the cross-walk (see Section 7.3.1). Many applications such as enhanced online map [102], road management [103], navigation systems for BVIP [104], and automated cars [87] have discussed this task. Images used to address this problem have been taken from a variety of perspectives: aerial [102,105], vehicle [87], and pedestrian perspectives [62].

The detection of crosswalks from natural scene images has to cater for many variations which complicates the task for trained models [106]. The specific challenges are:

- Crosswalks differ in shape and style across countries.
- The painting of crosswalks may be partially or completely worn away, especially in countries with poor road maintenance practices.
- Vehicle, pedestrians, and other objects may mask the crosswalk.
- Strong shadows may darken the appearance of the crosswalk.
- The change in weather and time when an image is captured affects the illumination of the image.

In addition to the lack of uniformity of crosswalks for detecting the presence and location of the crosswalk, BVIP need to be able to determine with precision the direction of the crosswalk on the road. If the system relies a camera to identify the crosswalk alignment in real-time, the captured images may only find part of a crosswalk or/and with a wrong angle. Several articles discuss these challenges. These papers employ a variety of approaches: traditional computer vision [106,107], traditional machine learning such as SVM [65], and deep learning algorithms [105,108]. The work of Wu et al. [106] concluded that deep learning outweighs traditional computer vision techniques in their comparisons. We analyse the deep learning works, based on grouping them as follows:

*Classification:* A pre-trained network VGG is used by Berrie et al. [105,108] to identify whether images contain a crosswalk or not. Tümen and Ergen [87] used a custom networked termed RoIC-CNN for the existence of crosswalks as a contribution to driven assistance research.

*Object detection:* With object detection, both the existence and location within a scene (image) is determined. Kurath et al. [102] employed a sliding window over an image to detect the crosswalk using an Inception-v3 model. Malbog [109] used MASK R-CNN to detect the crosswalk. This model outputs are bounding box, mask, and classification score.

*Segmentation:* Yang et al. [104] used a CNN semantic segmenter to detect a crosswalk and other objects from the road, where segmentation builds upon object detection by providing a precise placement, shape and scale of the crosswalk within a scene.

*Location detection:* detecting location of crosswalks is critical for the BVIP to determine a safe place to cross the road. Yu et al. [62] presented a modification on MobileNetV3 to detect the start and endpoint of a crosswalk.

*Datasets in Crosswalk Detection Research:* In Table 3, we list the datasets used in this task by researchers for modelling training and/or evaluation, including their availability to other researchers. The table highlights the diversity and coverage of used datasets. It describes the perspective, number, and coverage area of captured images.

Looking at the datasets in Table 3, we note that each dataset contains just one type of crosswalk (zebra crosswalk), and thus there are various shapes of crosswalk which are not included. This limits the generalisability of models generated from the associated research works. Only the Pedestrian Traffic Lane [112] dataset contains the geographic location of crosswalks, and thus is the only one currently suited to enriching maps with crosswalk locations. Most datasets do not cover the various crosswalk challenges (painting can be fading away, objects partially occluding it, etc.). The majority are local datasets and are not published for general use.

### 5.4. Sidewalk Detection

For BVIP, a sidewalk is a critical street component, as it is the safest area to walk on. Sidewalk detection is a task in an environment mapping phase, where it is required to build a comprehensive map based on sidewalks. This map helps in producing precise instructions for BVIP [113]. In BVIP navigation systems literature, sidewalk detection was discussed as an obstacle avoidance task where the navigation system detects them to avoid falling [47,60].

### 5.5. Public Transportation Information

We deem public transportation information as relevant to the mapping environment phase to support users who may wish to include public transport into their journey. Before using public transportation means, there are various types of information that need to be gathered such as the locations of public transportation stations or stops [114], accessibility information of stations and stops [115] and schedule of routes [116]. This level of information is relevant for the route selection task (see Section 6.2). Some of these details are available through applications or on the internet but not in a form that is easy to use by BVIP [117]. We suggest that this area needs to be recognised as a component to be deployed in an environment mapping application, with public transport information included as part of map enrichment.

### 5.6. Discussion of Environment Mapping Research

Having reviewed the levels and types of research approaches being undertaken in various aspects of environment mapping, we now take a summary view of the area.

The information and locations of PTLs, intersections, sidewalks, crosswalks, and public transportation need to be involved in maps for the benefit of BVIP undertaking a journey. The available work in intersection detection to date does not cover all types of intersections. The binary classification approach defines only the existence or not of an intersection. In addition, the accuracy of a multi-classification approach (six or seven types) is very low. While the direction of detecting a road before an intersection classification has a promising accuracy that ranges between 81.8 % 100 %, it only detects three types of intersection, which is not enough. These approaches do not define the location of a junction, which is critical in the environment mapping phase. In contrast, the object detection approach can detect the location of an intersection with 0.86 F1-score from map tiles. This location is on the image, but it can in theory be projected to the real location.

The crosswalk detection task has a variety of works using deep learning based computer vision approaches including classification, object detection, segmentation, and location detection. The environment mapping stage is more sophisticated than detecting the absence or existence of crosswalks. Therefore, appropriate directions are object detection, segmentation, and location detection approaches, as in theory they can all define crosswalk location. Only the location detection approach was tested for defining a start and end point of a crosswalk with an average angle error of 6.15° [62]. To the best of our knowledge, no paper discussed different shapes of the crosswalks (see Table 3).

### 5.7. Future Work for Environment Mapping

The environment mapping phase as a pre-stage for BVIP navigation needs to be addressed as a key area of BVIP navigation systems research. Approaches from other domains such as driver assistance and autonomous vehicles can be built upon to produce maps for BVIP navigation. Looking at the various approaches of intersection and crosswalk detection, object detection approaches hold promise for determining the type and location of each street component.

## 6. Journey Planning

Once the main components of an urban environment have been used to provide enriched maps (see details in Section 5), these maps will be used in the journey planning phase. The journey planning phase is used to plan the route to the user’s destination before starting their journey, helping the user to choose the optimal route, and providing a complete overview of the route before starting the journey. The following section will discuss research in support of journey planning in detail. The relative merits of the journey research approaches are then provided at the end of this section.

### 6.1. Localization

In the planning stage, a user has two options (1) obtain directions between two locations and (2) to obtain directions between their current location and destination. In the first option, the user will define a start and destination location. In the second one, the localization task is used to define their current location. Localization is an essential task in a variety of domains: robot navigation [118], automated cars [118], and BVIP navigation systems [23,80]. For BVIP, the precision of localization is significant because it affects the quality of instructions that are provided by a navigation system. The approaches of other applications are not enough for the safety of BVIP [59,113].

Indoor and outdoor localization systems employ different system architectures. Indoor approaches, such as radio frequency identification tags [119], active radio-frequency identification technology [24], and Bluetooth beacons [74,120], are not suitable for outdoor environments because they have a localized infrastructure that does not scale to outdoor. We identify two approaches to outdoor navigation systems, both of which are relevant to BVIP Localization. *Global Positioning Systems (GPS)* are employed in assistive outdoor navigation systems to receive data about the location of the user from satellites [22,23,54,55,56,59,75,80]. Typical GPS accuracy, in the range 20 metres, needs to be supplemented for pinpointing more fine grained location to support BVIP [73]. They employed an external GPS tracker to define the location of the user using a u-blox NEO-6M chip with a location accuracy of less than 0.4 m. A second approach is *image-based* positioning systems. This approach defines a location of a user by querying a captured image in a dataset that contains images and location information [36,37,38,58]. V-Eye [39] used visual simultaneous localization and mapping (SLAM) and model-based localization (MBL) to localize the BVIP with a median error of approximately 0.27 m.

### 6.2. Route Selection

After defining a journey start point, the optimal route(s) from start point to destination is determined during route selection, allowing for distance, safety and considerations of the BVIP base. Although this task is very important for BVIP, there is a limited amount of research to address it from the perspective of this user group [121,122]. Most BVIP outdoor navigation systems used available path finding services, such as QQMap [80], open source route planner [55] and BaiduMap [54], without personalised selection of the shortest path with allowance for the BVIP’s preferences. We suggest that is related to the issue of lack of street market BVIP relevant information on maps (like traffic lights, sidewalks, etc.)—all of which are needed to choose the best path for our user base.

Route selection consists of pedestrian routing and public transportation as sub-tasks (read Section 7.4). Public transportation as part of journey planning does not appear in the literature [32,78,79] therefore, the focus of this section is on pedestrian routing. This problem of route selection problem is a significant task for navigation of vehicles [123] or pedestrians with and without disabilities [122].

Route selection algorithms divide into two approaches, namely *static* and *dynamic* approaches, depending on their consideration or not of the time during the day (rush hour, morning, evening, etc.) [123]. The problem of route selection is solved in two steps. As a general approach, a graph is built first, including nodes, edges that link between nodes, and weights to evaluate each segment. Second, the routing algorithms step chooses the best route, allowing for predefined criteria assessed against weighted routes derived from the map [10].

In the literature, route selection has different terminology such as wayfinding, route planning, route recommendation, and path planning. Analysing the literature, we group the routing selection algorithms approaches into two groups. *Simple Distance criteria:* in this approach, graph weights depend only on the distance between nodes, so the routing algorithms choose the shortest path. Different routing algorithms are employed for this problem, such as Dijkstra’s algorithm [74] and particle swarm optimization strategy [124]. Secondly, we noted a *Customised Criteria* approach, where graph weights determine the accessibility of each edge and the distance between nodes to choose the optimal path of the user. Cohen and Dalyot [121] used information about length, complexity, landmarks, and way type from Open Street Map to build a network-weighted graph and used a Dijkstra algorithm to choose the best route. Fogli et al. [125] depended on using accessibility information (manually gathering) and Google Maps services to navigate disabled people.

We also reviewed orientation systems for other disabilities. For wheelchair users, Wheeler et al. [126] presented a sidewalk network that has accessibility information (width, length, slope, surface type, surface condition, and steps of each sidewalk segment), and a Dijkstra algorithm calculated the best road depending on that information. Bravo1 and Giret [122] constructed a wayfinding system that depends on the user profile (the type of disabilities) to find the best route according to each disability.

For BVIP route planning, we suggest that a customised criteria approach is required for suitable journey planning, utilising the information generated from the environment mapping phase in addition to accessibility information.

### 6.3. Discussion of Journey Planning Research

Looking at localization, GPS accuracy provides a precise location within 10–20 m [24], which is not as precise as that ideally required to pinpoint the exact location of BVIP. In addition, GPS is further affected by high buildings in crowded cities. On the other hand, the alternative approach using image-based localization reaches a median error of approximately 0.27 m. It requires enormous effort to collect local images with location information. Image-based depends on the ability of a blind user to capture a stable image to query over the image dataset. At this point in time, these data gathering and usability issues render the image-based approach unsuitable for BVIP Localization.

Looking at the research related to the route selection, disabled people require enjoyable, safer paths that are appropriate to their needs (fewer turns, more traffic lights, and so on) rather than the routes selected primarily on distance [127]. Therefore, customised criteria are considered a more promising approach than the simple distance based approach. We also noted that most navigation systems used the centre of the street (centre lines), and this negatively affects the accuracy of instructions for pedestrian navigation—particularly for BVIP [113]. While dynamic approaches depend on accessibility information, which increases user confidence about suggested routes, these approaches are not currently used in most navigation systems for BVIP [80]. Accessibility information plays a significant role in dynamic approaches but most of it is gathered manually [121,125]. Although most of the navigation systems for BVIP used the Dijkstra algorithm, the time response of this algorithm limits its suitability as the best option [128], especially on a large map. Finally, we note that navigation systems for BVIP did not incorporate public transportation into the journey planning phase.

### 6.4. Future Work for Journey Planning

For localization, the approach of using the external GPS tracker is suitable to define the location of the user, as used by Meliones et al. [73]. As per the previously stated pre-requisite for route selection, there is a need to build a system that can gather accessibility information automatically. We also identify that further investigation is needed to discover the most suitable algorithm for routing selection problems in terms of time response. Finally, we suggest building a navigation system that includes routing selection in any mode (walking or using public transportation) and using dynamic routing selection approaches to help BVIP in choosing the preferred route.

## 7. Real-Time Navigation

Having planned a journey and selected a route, the BVIP then needs support to detect dynamic factors in real-time during their pedestrian journey. Looking at Figure 1, this consists of understanding their surroundings, avoiding obstacles, crossing a road and using public transportation. In this section, the research efforts in support of these BVIP real-time navigation tasks will be presented in detail. The research discussion will be presented towards the end of the section.

### 7.1. Environment Understanding

The environment understanding task is about enabling the BVIP to perceive their physical surroundings in real-time. It includes enabling the BVIP to read signage and to gain an understanding of the immediate surroundings.

#### 7.1.1. Signage Reading

For understanding an environment, a user needs to understand what is happening around him/her. This task is concerned with enabling BVIP to be aware of the existence of, and to read, signage on the street [40,41]. This task is significant and it can alert to dynamic factors that are not captured on maps loaded with static information. Examples of such signage are those for closed road signs during maintenance or an area of construction work. The ability to perceive and use this type of signage is an important safety and confidence factor for BVIP, even on familiar routes. It was discussed in just two navigation systems works, as shown in Table 1).

#### 7.1.2. Surroundings Understanding:

BVIP need to understand their surroundings to interact with their environment. A typical scenario is BVIP walking in the street when an unexpected noise is perceived. The user needs to determine what is happening and whether/where they should continue walking via their planned route. Typical scenarios might be an accident, a broken water pipe, or encountering unexpected construction works along the road.

There are several research approaches used to help BVIP to interpret their immediate environments, such as scene recognition [58], multi-object detection [42], and scene caption [43]. Scene recognition is about classifying the image into pre-defined classe [58], while multi-object detection is to detect multiple objects on a single image [42]. Scene caption is considered the most suitable in this case, as it describes objects in context (environment) and their relation in sentence [129]. The task of understand surroundings is included in just four navigation systems, as shown in Table 1.

### 7.2. Obstacle Avoidance

In a real-time navigation phase, avoiding obstacles represents a continuous challenge for the BVIP. This task is about helping BVIP to avoid collisions with street obstacles, static or moving, at ground or raised level—so as to minimize injury, distress and reduction in confidence. The traversable area detection and obstacle avoidance are two sides of the same coin. While traversable area detection determines the area where a user can walk safely [130,131,132], an obstacle avoidance task detects the location of obstacles and assists the user in avoiding them [25].

BVIP need to know more than simply where the traversable area of a sidewalk is [130,131,132]. While the ground may be empty and traversable, there may be other kinds of obstacles that prevent walking safely, such as head, chest, and knee level obstacles. Consequently, framing safe navigation as an obstacle avoidance task is a more complete problem approach for BVIP navigation, than traversable area detection. Obstacle avoidance has become a high active research area in recent years, across robot navigation systems [133], BVIP navigation systems [23,28], and autonomous vehicles [134] research domains. To explore it more fully, we identify two groupings in the research approaches used. We also investigate the datasets used in support of the research, given the extent and role of datasets used in the domain.

We group obstacle avoidance approaches into the following groupings: *Obstacles Detection* indicates the existence of an obstacle or not, as opposed to identifying the nature of the obstacle. Cardillo et al. [33] and Pisa et al. [34] used radar in a conventional cane to detect obstacles. Kiuru et al. [35] presented a wearable device with a built-in radar to detect obstacles. Kaushalya et al. [22], Meliones et al. [73], and Sohl-Dickstein et al. [30] used an ultrasonic sensor to detect obstacles. Jeong and Yu [25] utilized seven ultrasonic sensors to detect the obstacles from the whole scene in front of the user and ground drop-offs. Patil et al. [31] utilized six ultrasonic sensors to detect obstacles on floor and knee levels and a wet floor detector sensor. Meshram et al. [23] used five ultrasonic sensors to detect obstacles at different levels, stairs’ types, and slops. They also utilized a liquid sensor to detect wet floors. Chang et al. [28] used an infrared transceiver sensor to detect the distance between users and aerial obstacles. Islam et al. [70] used three ultrasonic sensors to detect obstacles on the left, right, and in front of the user. They supplemented this with an ultrasonic sensor and a CNN model to detect the pothole. Rahman et al. [27] utilized three infrared sensors to detect right, left, and front obstacles. They calculated the distance between obstacles and a user by a triangulation algorithm. In contrast, a Microsoft Kinect camera was used to detect obstacles by Song et al. [67]. Martinez et al. [71] used a stixel segmentation algorithm with some modification to detect obstacles. Depth images were used to detect obstacles and define the distance between obstacles and a user, then depend on fuzzy logic to avoid obstacles [75].

All of these various works aim to detect the existence of an obstacle. Our second category, *Obstacles Recognition* aims to identify the type of object that is causing the obstacle. Poggi and Mattoccia utilized [50] an adapted LeNet architecture to recognize the nearest obstacles. DeepLabV3 is a semantic segmentation used to define 15 obstacles, such as a sidewalk, pole, building [60]. FuseNet generated semantic images to use with RGB and RGB-D images to provide walkable instructions for the user [44]. Duh et al. [39] and Yang et al. [47] used semantic segmentation to recognize obstacles. While Lin et al. [61] switched between Faster R-CNN and YOLO on different modes, Joshi et al. [68] used YOLO-v3. Chun et al. [26] used laser (LiDAR) sensor measures to define the types of hazards (staircase, ramp, drainage, pothole, and step).

Mocanu et al. [76] utilized a smartphone video camera to detect, track, and recognize obstacles. They also used an ultra-sensor to detect the distance between a user and obstacles which is a useful addition in the context of BVIP. Younis et al. [45] utilized MobileNets SSD to detect an object type and location. They then applied a Hungarian algorithm to track multiple objects, and a neural network to classify the level of hazard, which is relevant to BVIP scenarios. Bai et al. [80] used PeleeNet to recognize the obstacles, and they presented an algorithm to detect the location and orientation of obstacles.

*Datasets in Obstacle Avoidance Research:*Table 4 presents a summary of the datasets, approaches and number of objects used for the obstacle avoidance task. The number of obstacles defines the number of covered objects in each dataset whether they were applied for a BVIP use case or not. As shown in the table, there is no dataset that defines all needed obstacles from BVIP’s perspectives [44,60,68]. Although Lin et al. [44] built a dataset with 6000 obstacles for BVIP’s usage, this dataset contains only low-lying obstacles. This table underlines the need to build a new dataset from BVIP perspective that cover objects on different levels.

### 7.3. Crossing the Street

In a real-time navigation phase, a user will need to cross a road from time to time. First, BVIP need to find and position themselves correctly at a safe crossing point. They then need, if at a traffic light, to wait for a green light to cross a road. In the following subsections, tasks that are needed to accomplish a crossing street mission in real-time safely and independently, as covered in Figure 1.

#### 7.3.1. Crosswalk Alignment

Pre-defining the location of crosswalks provides the BVIP with accurate instructions to reach a crosswalk location (see Section 5.3). When a visually impaired person reaches a crosswalk, (s)he needs to be aligned or positioning correctly at the crosswalk so as to cross road safely, within the zone of the crosswalk boundaries [51]. Images are needed to align the user with a crosswalk in real time. The image for a crosswalk area can be captured by a user [62], an automatic image shooting mechanism [77], or from satellite images [65]. It is a challenge for a visually impaired person to capture an image, with the capture method suffering from instability [77].

#### 7.3.2. Pedestrian Traffic Light Recognition

The second task under crossing the street is the recognition of pedestrian traffic lights (PTLs). Recognition of PTLs is a significant task for BVIP to define when it is safe for them to cross a road [62]. In general, there are two types of traffic lights, namely pedestrian and vehicle traffic lights. Pedestrian navigation systems are interested in PTLs [62]. In contrast, driver assistance and autonomous car systems are concerned with vehicle traffic lights [138]. Rothaus et al. [139] addressed the challenges of detection of traffic lights when using a smart phone, but these challenges apply to any real-time image capture system: Firstly, PTLs have different shapes in different urban areas, within or across countries. The scale of PTLs will be different according to the distance between pedestrians and lights (different size). Vehicles and other objects may physically block a light if they are positioned across the crossing point. There may be multiple PTLs in a single scene (i.e., image), but the user is concerned with using the right light to get them across the relevant piece of road they need to cross on their journey. Sometimes, captured images and videos may not be stable, and can lack consistency on angles and quality. Detection algorithms must be robust enough to deal with low qualities and resolutions of images and videos. In images, there are variations in illumination from day or night and in weather conditions. There are limitations on memory space and computational power. All of these factors present challenges to producing stable, generalisable algorithms.

Unsurprisingly, the recognition of pedestrian and vehicle traffic lights overlap in their approaches. Since there was a stronger focus on vehicle traffic lights in the research literature than on PILs, we will explore both of them. We suggest however that challenge for PTLs is potentially bigger. For instance, images captured via a driver assistance system/autonomous car, where the camera is typically mounted, will be more stable than those captured via a wearable or handheld camera at a BVIP navigation system.

While diversified sources of data such as RADAR and LIDAR are used to detect the existence of traffic lights, computer vision-based approaches are required to identify traffic lighting colours/status [140]. Before using deep learning, traditional computer vision techniques (color segmentation and shape segmentation) and traditional machine learning algorithms (SVM and tree-based model) were used. The comparison between classical approaches, traditional machine learning, and deep learning indicates that deep learning approaches offer the most promising and state of the art direction [62,140,141]. Deep learning can extract better features in real-time conditions and learn better feature combinations to handle difficult situations such as over-exposure, color distortions, and occlusions. Automatically detecting traffic lights breaks down into three areas: traffic light detection (existence), traffic light state classification (light status), and tracking traffic light (help during time limitation or occlusion) [140]. The output of traffic light detection task is bounding boxes around traffic lights, while traffic light state classification’s output is the state of the traffic light. In a tracking stage, a previous state is tracked [140]. While many articles covered traffic light detection and traffic light state classification [53], traffic light tracking is typically not included [64], and we note this gap. We present previous work as two groups, based on whether they combine traffic light detection and state classification into one step, whether they treat this as a two-stage process, where each is done using a separate network.

*A one-stage class:* Li et al. [53] used a simple CNN network to detect and classify traffic light. Ash et al. [64] presented a system that detects a PTLs status, and it tells a user to walk or stop. They did two experiments, using a Faster RCNN with a Kernelized Correlation Filters (KCF) tracker, and a YOLOv2 based network. Yu et al. [62] presented a mobile phone application to help BVIP to cross the road. It modified the MobileNetV3 by utilizing depth-wise separable convolutions, inverted residuals and linear bottlenecks, and squeeze-excite layers. The Faster R-CNN model was utilized to define the bounding box and its score [142,143]. Ghilardi et al. [63] used alternative CNN architectures for the same purpose of traffic light detection and state classification. To detect small traffic lights, some architectures of deep learning are presented. Lee and Kim [144] presented architecture that contains three main components, encoder, decoder, and detector. The output is bounding boxes, confidences, and class probabilities. In addition, they used a focal regression loss to make a balance between easy and difficult examples, so the efficiency of the system increased. Muller and Dietmayer [145] introduced an improvement over the single shot detection algorithm to detect small traffic lights. First, they replaced VGG with an Inception v3 network to increase the speed and accuracy. Secondly, they presented an enhancement on prior boxes to stride smaller in later layers and used non-maximum suppression to prevent detect an object more than once. Finally, they detected the state of traffic light by adding a new branch for the basic network.

*Two-stages class:* in this second approach, each task (detection and traffic light state classification) was achieved in two separate steps. Hassan and Ming [146] utilized a classical color segmentation method to detect the PTL, then used CNN to recognize the status of PTL. Ouyang et al. [147] built a real-time system to detect traffic light. First, they utilized Gaussian Filter, Top Hat Morphology, OTSU algorithm, and HIS transformation to recognize a region of interest (ROI). Second, they built a new CNN architecture to classify each ROI. Gupta1 and Choudhary [148] used Faster R-CNN to detect a location of traffic light and a bounding box, feeding the result to a VGG network to generate a feature vector. They then used this with Grassmann Manifolds to classify the bounding box.

To recognize small traffic lights on images, Lu et al. [149] used a Faster R-CNN network to detect ROIs in an image. Then, ROIs were fed to another Faster R-CNN that detected a bounding box of an object and its confidence. Behrendt et al. [150] used a modified Yolo algorithm to detect traffic light, utilized a small CNN network to recognize the status of traffic light, and then tracked it by using an odometry-based motion model.

To detect traffic light at different times and various weather conditions, Zhang et al. [151] suggested detecting ROIs by color and shape segmentation, then using DNN to classify each ROI. Saini et al. [152] used a color segmentation, shape, and area analysis to define traffic light candidates. Then, they utilized Maximally Stable Extremal Region for structure localization. After that, they used histogram of oriented gradients (HOG) as a descriptor for each candidate and SVM to decrease the false-positive detected traffic lights. Finally, the status of the traffic light is classified using CNN. *Auxiliary map based:* Some research work rely on information from a map to detect traffic light. John et al. [153,154] built a salience map that contains a GPS location of a car and ROI of the nearest traffic lights in good illumination conditions. They used a salience map in low illumination conditions, to detect the ROI of the traffic lights [153,154]. Then, a CNN is used to detect the traffic light status. While the previous work used map information to decrease a search area, Possatti et al. [155] used information on a pre-constructed map to define a relative traffic light to the vehicles, as one image may contain more than traffic lights. The offline map was built by detecting traffic lights locations and defined manually the relevant one for each trajectory.

*Datasets in Pedestrian Traffic Light Recognition Research:* Datasets are used throughout traffic light detection research works to support the training and testing of robust deep learning models. Table 5 defines used datasets for training a PTLs recognition model in previous works. It includes details about each dataset, such as the number of images, conditions, coverage area, and availability. Hassan and Ming [146] used three groups of images: 200 images for HSV threshold selection, 5000 images for classifiers training, and 400 images for testing. Looking at Table 5, we note that the number of images in each dataset is very limited. Datasets were captured in one country (one shape), which will affect the generalisability of the resultant model to cater for a range of PTL shapes. To the best of our knowledge, there is no dataset covers all challenges that are needed to ensure the robustness of a model, such as illumination, day and night, variation in scale, weather conditions. Finally, we note that most of the datasets are not available online.

### 7.4. Using Public Transportation Systems

During real-time navigation, a user often needs to use a public transportation system for long journeys. Developing assistive navigation systems that support different modes of available public transport, such as bus and metro, will increase the independence and take-up of such systems by BVIP.

**Figure 3:**
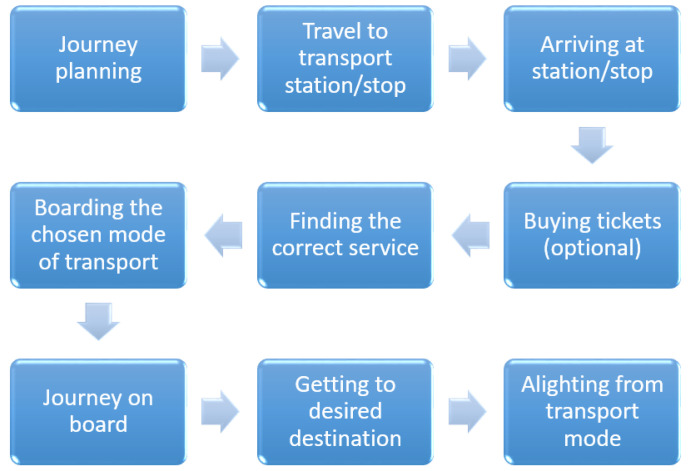
Journey cycle on public transport for BVIP (modified from Low et al. [117], Lafratta [157], and Soltani et al. [158]).

The tasks of using public transport systems consist of multiple steps, as shown in Figure 3. Lafratta [157] and Soltani et al. [158] discussed a journey cycle for use of public transportation by disabled people while Low et al. [117] presented a journey cycle for BVIP in London. We generalized the journey cycle by Low et al. [117] to fit different scenarios in various countries by adding a ’buying tickets’ stage, which is mandatory in Lafratta [157] and Soltani et al. [158].

Each step on this task merits consideration in any study to determine the needs of BVIP across multiple contexts. For instance, ’ Finding the correct service’ step at the bus stop is about catching the right bus for the destination [32,66,78,79] when it arrive at a bus stop. In contrast, this step is more complicated in the airport [159] or large scale train station [114].

### 7.5. Discussion of Real-Time Navigation

Having presented research activity by task, we now discuss the overall research activity to support the real-time navigation phase for BVIP. The majority of aid systems did not include signage reading, surroundings understanding tasks (see Table 1). For a PTL recognition task, there are some limitations in previous works. Firstly, they search on the whole image which increases the number of false positives. Secondly, they did not have an approach to define the relative traffic lights on the image. These drawbacks are solved in autonomous cars domain by using an auxiliary map [153,154,155]. However, building an auxiliary map is time consuming [153,154,155], and in reality, the construction of an auxiliary map is not practical. Finally, most of the challenges for PTL detection and state classification are not solved, as set out in Table 6.

In contrast, a considerable number of published research exists for obstacle avoidance (see Table 1). There are many types of hurdles that face BVIP, such as aerial, knees, ground, static, and dynamic obstacles. However, the various obstacle avoidance systems each cover just a limited number of hurdles. In addition, where the problem is treated solely as an obstacle detection approach, the types of objects are not dealt with, which limits the usefulness for BVIP. For this reason, obstacle recognition is a promising approach, gleaning richer information about obstacles. As shown in Table 4, distinct approaches, such as object detection, semantic segmentation, were used. However, response time and size of models are still limiting factors that need to be considered during the implementation of this approach [60].

A pedestrian traffic light recognition problem and an obstacles avoidance problem are solved using different approaches. These approaches apply different matrices to compute efficiency which prevents comparison between them. In addition, not all approaches are available online to enable a fair comparison.

There is no general solution to support using different public transportation means during BVIP navigation. Importantly, there are recent surveys [117,160] done to explore gaps and limitations in this area. These surveys declare the limited work done in this research area.

### 7.6. Future Work for the Real-Time Navigation Phase

The future work in a signage reading area can be inspired by work done in a scene text detection and recognition area [161,162,163]. The purpose of this work is general and has many practical applications, such as assistance for BVIP, text translation, robotics, autonomous driving. To the best of our knowledge, no research work has discussed adding this feature to outdoor navigation systema for BVIP.

The backbone of building scene caption is the existence of datasets. While there are different datasets available for this task [129], none of them were captured from the BVIP perspective. In addition, the available captions for these datasets were not applied or verified as being sufficient for scene description for BVIP.

For obstacle avoidance, there is a need to build datasets that include different types of obstacles according to the typical scenarios and needs for BVIP navigation. Furthermore, obstacle avoidance needs more analysis to define an appropriate action depending upon the obstacle type. For example, if there is a tree branch alongside the sidewalk, what action should the BVIP take? We suggest using a method to evaluate the situation (level of hazard) [45], then generating a compatible instruction [44]. Additionally, we suggest an obstacle avoidance system that depends on sensors to continuously detect obstacles and to use a camera from time to time where a scene description is needed, so as reduce consuming power. In addition, when there is a complicated situation, we suggest utilizing a camera to recognize the type of obstacles and handle them.

We suggest utilizing aerial images to detect a crosswalks’ location, as mentioned in Section 5.3, then provide a user with instructions to reach it. For an alignment task, a visually impaired person is directed to capture a real image when reaching the crosswalk location. This will guarantee more safety, reduced power consumption and more stable images.

For the PTL recognition problem, a large and diverse PTL dataset is needed. It must include images from different countries, cover various illumination conditions, day and night, variation in scale, distinct weather conditions, etc. We also need to build a robust model that takes into consideration the challenges that we mentioned in Table 6. In our opinion, an auxiliary map-based is considered the best direction to follow. It can help in decreasing a search area on image and help in low illumination conditions. In addition, it can define the relative traffic light, which is a significant challenge for BVIP. However, a practical method to building this map requires further investigation.

## 8. Feedback and Wearability of Navigation Systems Devices

To date, our focus has been on the research work underpinning each of the functional tasks of navigation system. Other important aspects for comparison include feedback, coverage (indoor or outdoor), portability (weight), cost, energy consumption, latency, user-friendly, etc. [8,15,25,76]. Both feedback and wearability as closely related to functionality, as a core part of device usage and design, so we points to principal research works in this area for use by the research community.

Feedback can be defined as the means used by the system to convey information to the BVIP. Aid systems use audio [6,29,32,69,164,165], haptic [25,166,167], or a combination of these two [50,168]. Using headphones (sound feedback) to receive information from the system has the disadvantage of blocking out other audio information for the user, affecting their perception of a surrounding [10,168]. This problem can be solved using bone-conducting headphones [169] which convey sounds through vibrations on cheekbone [47,132]. Feedback requires further investigation in the amount and meaning of information that will be sent to the user [10,168].

The wearability of a device is a key consideration during the design stage, defining how the device will be attached to or carried by the user, with a focus on keeping the user as flexible and unrestricted as possible. The options for wearability include (1) a wearable device where a user can wear the device in a natural way, such as a waist belt or glasses [41,75,76,170], (2) a hand-held device which the user can held in their hand such as a smart phone [25,54], and (3) a combination between these two [26,44]. Genuinely wearable devices outweigh hand-held devices, as the user’s hands are free [16] and the stability of the captured image is higher.

## 9. Applications and Devices

Whilst the largest focus of this review is the active research work in BVIP and overlapping navigation systems work, we also include an overview of the applications and devices available to be used by BVIP in real life. Table 7 presented a detailed and comprehensive comparison between them. For each one, we declare a name, components, features, feedback/wearability/cost, and limitations. Components are the physical hardware components available to the user, while features summarise the functionality offered by the device. The output will be stated in a feedback column. A wearability column describes the carry mode of the device. Finally, for each one, the disadvantages are defined in the weak points column. These application and devices can be divided according to carry mode into wearable and handheld categories.

### 9.1. Handheld

A handheld is a device, or application that is held in a user’s hand. UltraCane [177], and SmartCane [173] are examples of handheld devices. All these devices are traditional canes, with enhancements added to detect all levels of obstacles.

WeWalk [174] provides users with a cane that contains sensors to detect obstacles on all levels and a mobile app for navigation guidance. It can control the mobile phone during a cane, so one hand will be free. Nearby Explorer [181] gives information about objects that the user points to, such as distance, height. PathVu Navigation [183] gives information only about obstacles that were informed about them by another user, so a user must use the traditional methods to detect other obstacles.

Aira [190] and Be My Eyes [191] are phone applications that provide support to BVIP in difficult situations, such as when lost or when faced with obstacles. These applications do not preserve user privacy.

### 9.2. Wearable

Some navigation applications or devices can be worn without occupying the BVIP hand. Wearable devices such as Maptic [171] and Sunu Band [188] do not discover obstacles on all levels, so a user must use other devices, such as a cane. However, Horus [175], Envision Glasses [179], and Eye See [180] do not provide users with navigation guidance.

### 9.3. Discussion

**Figure 4:**
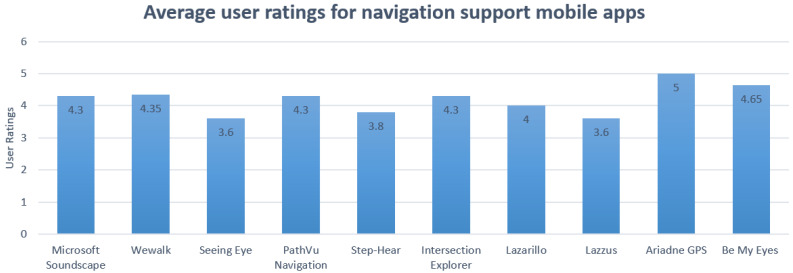
User experience for navigation support mobile apps.

At present, the available applications and devices do not support all mandatory tasks for navigation activity. The majority of aid devices and applications support obstacle avoidance and guidance tasks (see Table 7). Although there are two means of feedback, the majority of applications provide feedback via audio. Using a headset for audio feedback raises the problem of blocking out other environmental audio sounds, but this can be solved using bone-conducting headphones. Most mobile application are free, while other navigation assistive devices are not. Wearable devices, although not yet common, have the advantage of being hands-free. Real end-users experiences with available applications and devices are very important. This kind of information is generally only available for mobile apps. We collected end-user ratings from Google Play Store and Apple App Store taking the average rating of each, as shown in Figure 4.

## 10. Main Findings

The principal finding of our review is that although development has been done in this field, it is still some distance from producing complete and robust solutions for BVIP navigation support.

The previous analysis of the environment mapping phase demonstrates that various annotations are needed to available maps. These annotations include safety critical information on the location of PTLs, intersections, sidewalks, crosswalks, and public transportation (review Section 5.6). Localization of BVIP needs to yield highly precise locations, and typical GPS accuracy is not adequate. Selection of the optimal route for BVIP is not about the shortest path. It is about an enjoyable, safe, well supported route appropriate to their needs (fewer turns, more traffic lights, and so on) [127]. Most of the navigation systems for BVIP do not discuss using public transportation. Accessibility information has a great role in routing selection task, while most of it is gathered manually (review Section 6.3).

Environment understanding is not included in the majority of aid systems. There is limited work done in the area of PTL recognition tasks. Each available obstacle avoidance system covers a limited number of hurdles, but it is not practical to use different systems at the same time to avoid each type of danger on the road. A more generalised obstacle avoidance system approach required.

No single BVIP application or device of those available are considered a comprehensive solution for BVIP (review Section 9.3). We also point out that differences exist in the terminology for the navigation systems area for BVIP (review Section 2.2).

### 10.1. Discussion

For benchmarking, a huge dataset(s) is required with a sufficient number of images for each type of intersection, crosswalk, PTL, sidewalk, scene, and obstacle. These images must be acquired under different conditions (illumination, shadow), various times (day and night), in different countries, with a diversity of conditions (objects partially occluding the crosswalks, shadows of other objects may be partially or completely darkening the road), and styles. The shortage of datasets not only influences the effectiveness of solutions for each task, it also means that there is no common way to compare solutions. Most algorithms are not available online to allow a fair comparison between current solutions. Apart from the the features and tasks for BVIP navigation systems already covered, other aspects such as wearability, feedback, cost, coverage, etc. need to be considered during the design stage. Users are reliant on these mobile devices when they are out walking, so energy consumption is a concern. A potential widespread disadvantage in real devices and applications is that the user may need to use more than one device to cover all of their initial needs.

Most of the presented navigation systems were not tested by end-users. Consequently, the status of user satisfaction regarding the services provided by research on BVIP navigation systems is unknown. This is a critical point that needs to be covered for two reasons. First, it will enhance research in this domain according to users’ opinions. Secondly, it will encourage manufacturing of prototypes that meet users’ requirements. For real applications and devices, user ratings are available only for mobile apps, see Figure 4.

### 10.2. General Comparison

Electromagnetic/radar-based systems were found to outperform sensor-based systems, both of which are mainly used on obstacles avoidance tasks, see Table 1. The high frequency in these systems corresponds to a smaller wavelength which in turn leads to compact, lightweight circuits. In addition, they can differentiate between near objects and detect tiny gaps and hanging obstacles [193].

Camera-based systems are affected by weather and illumination conditions, but provide more detail about obstacles such as shape and color. The advantage of smartphone-based systems is that one device contains different useful components that are need for navigation tasks, such as camera and GPS. These technologies are used in the majority of mandatory tasks required by BVIP navigation systems, see Table 1.

## 11. Conclusions and Future Work

Our review presents a comprehensive survey of outdoor BVIP navigation systems. Our paper improves on previous surveys by including a broad overview of the area and detailed investigations about research completed for each stage. This provides a highly accessible way for other researchers to assess the scope of previous work done against the task area of interest—even if they are not concerned with the end-to-end navigation view. In each task, we investigate the algorithms used, research datasets, limitations, and future work. We clarify and explain the different terminology used in this field. In addition to research developments, we provide details about applications and devices that help BVIP in urban navigation.

In summary, more work is needed in this field to present a reliable and comprehensive navigation device for BVIP. We also emphasize the need to transfer learning between other domains to this domain, such as the domains of automated cars, driver assistance and robot navigation. The design of navigation systems should consider other preferences, such as wearability and feedback. Deep learning-based methods described will require real-time network models so power consumption will be a practical concern, relative to the type of device it is running on. For example, the feasibility of running real-time obstacle detection via wearable camera device needs to be determined, for the various methods in the literature- but for now, most of the research is “lab-based”, focusing on achieving accurate results, rather than dealing with deployment issues of power consumption and device deployment. These issues will need to be addressed as more complex deep learning solutions become the state of the art for wearable vision support systems. 

## Figures and Tables

**Table 2:** Intersection datasets.

Datasets Name	Capture Perspective	Number of Images	Coverage Area	Available On-Line	Paper	Year
Tümen and Ergen dataset [87]	Google street view (GSV)	296 images	N/A	No	[87]	2020
Saeedimoghaddam and Stepinski dataset [86]	Map tiles	4000 tiles	27 cities in 15 U.S. states and captured the maps of different years	No	[86]	2019
Part of Oxford RobotCar dataset [94]	Vehicle	310 sequences	Central Oxford	No	[84]	2017
Part of Lara [95]	Vehicle	62 sequences	Paris, France	No	[84]	2017
Part of Cityscapes dataset [96]	Vehicle	1599 images	Nine cities	Yes	[92]	2017
Kumar et al. dataset [88]	Grand Theft Auto V (GTA) [97] Gaming platform	2000 videos from GTA and Mapillary [98]	-	No	[88]	2018
Construct videos from Mapillary [98]	Vehicle	2000 videos from GTA and Mapillary [98]	6 continents	No	[88]	2018
Construct dataset form KITTI [99]	Vehicle	410 images +70 sequences	City of Karlsruhe, Germany	No	[93]	2019

Legend: (N/A) information not available.

**Table 3:** Crosswalk datasets.

Datasets Name	Perspective	Number of Images	Type	Conditions (Day/Night, etc.)	Coverage Area	Available On-Line	Paper
GSV dataset	GSV	657,691	Zebra	Crosswalk lines may disappear, Crosswalks are partially covered, shadows affect the illumination of the road, different styles of zebra crosswalks	20 states of the Brazil	No	[108]
IARA	Vehicle	12,441	Zebra	Capture during the day	The capital of Espírito Santo, Vitória	Yes	[108]
GOPRO	Vehicle	11,070	Zebra	N/A	Vitória, Vila Velha and Guarapari, Espírito Santo, Brazil	Yes	[108]
Berriel et al. dataset [105]	Aerial	245,768	Zebra	Different crosswalk design, and different conditions (Crosswalk lines may disappear, Crosswalks are partially covered and so on)	3 continents, 9 countries, and at least 20 cities	No	[105]
Kurath et al. dataset [102]	Aerial	44,705	Zebra	N/A	Switzerland	No	[102]
Tümen and Ergen dataset [87]	GSV	296	Zebra	N/A	N/A	No	[87]
Part of Mapillary Vistas dataset [110]	Street level	20,000	Zebra	Images captured with different camera in different weather, season, point of view and daytime	6 continents	Yes	[104]
Cheng et al. Dataset [111]	Pedestrian	191	Zebra	N/A	N/A	Yes	[104]
Pedestrian Traffic Lane [112]	Pedestrian	5059	Zebra	N/A	N/A	Yes	[62]
Malbog dataset [109]	Vehicle	500	Zebra	Images captured in the morning and afternoon periods	N/A	No	[109]

Legend: (N/A) information not available.

**Table 4:** Obstacle avoidance datasets.

Datasets Name	#Num of Images	Number of Obstacles	Approach	Paper	Year
Shadi et al. dataset [60]	2760 images	15 objects for BVIP’s usage	Semantic Segmentation	[60]	2019
Cityscapes dataset [96]	5k fine frames	30 objects	Semantic Segmentation	[39]	2020
Part of Scannet dataset [135]	25k frames	40 objects	Semantic Segmentation	[44]	2019
Cityscapes dataset [96]	5k fine frames	30 objects	Semantic Segmentation	[44]	2019
RGB dataset	14k frames	6k objects for BVIP’s usage	Semantic Segmentation	[44]	2019
RGB-D dataset	21k frames	6k objects for BVIP’s usage	Semantic Segmentation	[44]	2019
PASCAL dataset [136]	11,540 images	20 objects	Object Detection	[61]	2017
Lin et al.dataset [61]	1710 images	7 objects	Object Detection	[61]	2017
Part of PASCAL dataset [136]	10k image patches	20 objects	Patch Classification	[76]	2016
Common Objects in Context (COCO) dataset [137]	328k images	80 objects	Object Recognition	[45]	2019
PASCAL dataset [136]	11,540 images	20 objects	Object Recognition	[45]	2019
Yang et al. dataset [47]	37,075 images	22 objects	Semantic Segmentation	[47]	2018
Joshi et al. dataset [68]	650 images per class	25 objects	Object Detection	[68]	2020
COCO dataset [137]	328k images	80 objects	Object Detection	[80]	2019

Legend: (N/A) information not available.

**Table 5:** Pedestrian traffic lights datasets.

Datasets Name	#Num of Images	Conditions (Day /Night, etc.)	Country	Available On-Line	Paper	Year
Li et al. dataset [53]	3693 images	N/A	New York City	No	[53]	2019
Ash et al. dataset [64]	950 color images, 121 short videos	Taken during daytime	Israel	No	[64]	2018
Hassan and Ming dataset [146]	400 images (HSV threshold selection) +5000 images (train) +400 images (test)	Variation in lights (HSV threshold selection) Different in distances from PTLs (test)	Singapore	No	[146]	2020
Pedestrian Traffic Lane [112]	5059 images	Variation in weather, position, orientation, and diverse size, and type of intersections	N/A	Yes	[62]	2019
Pedestrian Traffic Light [156]	4399 images	N/A	Brazilian cities	Yes	[63]	2018
Part of Mapillary Vistas dataset [110]	20,000 images	Images captured with different camera at different weather, season, point of view and daytime	6 continents	Yes	[104]	2018
Cheng et al. dataset [52]	17,774 videos	N/A	China, Italy, and Germany	Yes	[104]	2018

Legend: (N/A) information not available.

**Table 6:** Traffic light challenges that have been solved in the research literature.

Paper	Year	Traffic Light Type	Different Shapes	Tracking	Detect Active Colour	Low Resolutions	Different Size	Stability	Illumination
[53]	2020	Pedestrian							
[64]	2018	Pedestrian		✔					
[146]	2020	Pedestrian					✔		
[62]	2019	Pedestrian					✔		✔
[63]	2018	Pedestrian							
[104]	2018	Pedestrian							✔
[147]	2019	Vehicle							
[148]	2019	Vehicle							
[144]	2019	Vehicle					✔		
[149]	2018	Vehicle				✔	✔		
[145]	2018	Vehicle					✔		
[151]	2017	Vehicle							✔
[152]	2017	Vehicle							✔
[155]	2019	Vehicle		✔			✔		
[153]	2014	Vehicle			✔				✔
[150]	2017	Vehicle		✔			✔		

**Table 7:** Real navigation devices and applications.

Name	Components	Features	Feedback/Wearability/Cost	Weak Points
Maptic [171]	Sensor, Several feedback units, Phone	(1) Upper body obstacles detection (2) Navigation guidance	Haptic/Wearable/Unknown	Ground obstacles detection not supported
Microsoft Soundscape [172]	Phone, Beacons	(1) Navigation guidance (2) points of interest information	Audio/Handheld/Free	Obstacles detection not supported
SmartCane [173]	Sensor, Cane, Vibrations unit	Obstacles detection	Haptic/Handheld/ Commercial	Navigation guidance not supported
WeWalk [174]	Sensor, Cane, Phone	(1) Obstacles detection (2) Navigation guidance (3) Using public transportation (4) Points of interest information	Audio and haptic/Handheld (weight = 252 g/0.55 pounds (The weight of the white cane is not included))/ Commercial ($599)	Obstacle recognition and scene description not supported
Horus [175]	Bone conducted headset, two cameras, battery and GPU	(1) Obstacles detection (2) Read text (3) Face recognition (4) Scene description	Audio/Wearable/Commercial (cost around US $2000)	Navigation guidance not supported
Ray Electronic Mobility Aid [176]	Ultrasonic	Obstacles detection	Audio and Haptic/Handheld (60 g)/Commercial ($395.00)	Navigation guidance not supported
UltraCane [177]	A dual-range, Narrow beam ultrasound system, Cane	Obstacles detection	Haptic/Handheld/ Commercial (£590.00)	Navigation guidance not supported
BlindSquare [178]	Phone	(1) Navigation guidance (2) Using public transportation (3) Points of interest information	Audio/Handheld/ Commercial ($39.99)	Obstacles detection not supported
Envision Glasses [179]	Glasses with camera	(1) Read text (2) Scene description (3) Help in finding belongs, detect colours, Scan bar-codes (4) Recognize faces, make calls,	ask for help and share context >Via audio/Wearable (46 g)/ Commercial ($2099)	Obstacle detection and navigation guidance not supported
Eye See [180]	Helmet, Camera, Laser	(1) Obstacle detection (2) Read text (3) People descriptions	Via audio/Wearable/Unknown	Navigation guidance not supported
Nearby Explorer [181]	Phone	(1) Navigation guidance (2) Points of interest information (3) User tracking (4) Object’s information	Via audio and haptic/Handheld/Free	Obstacles detection not supported
Seeing Eye GPS [182]	Phone	(1) Navigation guidance (2) Points of interest and intersections information	Audio/Handheld/Commercial	Obstacles detection not supported
PathVu Navigation [183]	Phone	Alert about sidewalk problems	Via audio/Handheld/Free	Obstacles detection and navigation guidance not supported
Step-hear [184]	Phone	(1) Navigation guidance (2) Using public transportation	Via audio/Handheld/Free	Obstacle detection not supported
InterSection Explorer [185]	Phone	Information about street and intersections	Audio/Handheld/Free	Obstacles detection and navigation guidance not supported
LAZARILLO APP [186]	Phone	(1) Navigation guidance (2) Using public transportation (3) Point of interests information	Audio/Handheld/Free	Obstacles detection not supported
Lazzus APP [187]	Phone	(1) Navigation guidance (2) points of interest, crossings and intersections information	Audio/Handheld/Commercial (one year license $29.99)	Obstacles detection not supported
Sunu Band [188]	Sensors	Upper body obstacles detection	Haptic/Wearable/ Commercial ($299.00)	Ground obstacles detection not supported
Ariadne GPS [189]	Phone	(1) Navigation guidance (2) Explore the map	Audio/Handheld/Commercial ($4.99)	Obstacles detection not supported
Aira [190]	Phone	Support by sighted person	Audio/Handheld/ Commercial ($99 for 120 min)	Very expensive and Not preserve privacy
Be My Eyes [191]	Phone	Support by sighted person	Audio/Handheld/Free	Not preserve privacy
BrainPort [192]	Video camera a hand-held controller, a tongue array	Object detection	Haptic/Handheld and wearble/Commercial	Navigation guidance not supported

## Data Availability

The study does not report any data.
